# Synergistic Effect of Newly Introduced Root Canal Medicaments; Ozonated Olive Oil and Chitosan Nanoparticles, Against Persistent Endodontic Pathogens

**DOI:** 10.3389/fmicb.2018.01371

**Published:** 2018-07-03

**Authors:** Mohamed I. Elshinawy, Lamiaa A. Al-Madboly, Walaa M. Ghoneim, Nehal M. El-Deeb

**Affiliations:** ^1^Department of Endodontics, Faculty of Dentistry, Tanta University, Tanta, Egypt; ^2^Department of Restorative Dental Sciences, College of Dentistry, King Khalid University, Abha, Saudi Arabia; ^3^Department of Pharmaceutical Microbiology, Faculty of Pharmacy, Tanta University, Tanta, Egypt; ^4^Biopharmacetical Product Research Department, Genetic Engineering and Biotechnology Research Institute, City of Scientific Research and Technology Applications, Alexandria, Egypt

**Keywords:** anti-biofilm, root canal, ozonated olive oil, chitosan nanoparticles, endodontic pathogens

## Abstract

This study was conducted to investigate the antimicrobial-biofilm activity of chitosan (Ch-NPs), silver nanoparticles (Ag-NPs), ozonated olive oil (O_3_-oil) either separately or combined together against endodontic pathogens. While testing the antimicrobial activity, Ch-NPs showed the least minimum inhibitory concentration (MIC) and minimum bactericidal concentration (MBC) values exerting eightfold higher bactericidal activity than O_3_-oil against both *Enterococcus faecalis* and *Streptococcus mutans* as well as fourfold higher fungicidal activity against *Candida albicans*. Antimicrobial synergy test revealed synergism between O_3_-oil and Ch-NPs against the test pathogens (FIC index ≤ 0.5). Ch-NPs was superior at inhibiting immature single and mixed-species biofilm formations by 97 and 94%, respectively. Both of O_3_-oil and Ch-NPs had a complete anti-fibroblast adherent effect. The safety pattern results showed that O_3_-oil was the safest compound, followed by Ch-NPs. The double combination of Ch-NPs and O_3_-oil reduced the mature viable biofilm on premolars *ex vivo* model by 6-log reductions, with a fast kill rate, indicating potential use in treating root canals. Therefore, the double combination has the potential to eradicate mature mixed-species biofilms and hence it is potent, novel and safe.

## Introduction

The primary goal of endodontic therapy is to eradicate microbial infection and promote periapical tissue healing. Endodontic infections are polymicrobial and made up of various microorganisms that differ between failed endodontic cases and primary endodontic infections (Nair, 2006; Tirali et al., 2017).

Gram-negative, black-pigmented anaerobes are typically associated with primary endodontic infections, whereas Gram-positive streptococci and enterococci, particularly *Enterococcus faecalis* and *Streptococcus mutans*, and *Candida albicans* are more common in failed or persistent endodontic infections (Peciuliene et al., 2000; Nair, 2006; Tirali et al., 2017).

In endodontic microbiology, biofilm-mediated infection is the primary cause of endodontic disease. Bacterial biofilm is made up of bacterial cell colonies that form an extracellular matrix substrate that adheres to organic surfaces. Persistent and chronic endodontic infections depend in part on the density, diversity and resistance of bacterial biofilms to both host defense mechanisms and antimicrobial agents. Other contributing factors are the complexity of the root canal anatomy including the lateral canals, ramifications and tubular nature of the dentin that harbors bacterial colonies (Kishen, 2010; Siqueira et al., 2010).

Chemomechanical debridement of root canals is reported to be effective for eliminating bacterial biofilm and disinfecting root canals (Kishen, 2010). However, the current intracanal medicament and irrigation solutions can be harmful and cytotoxic to the host tissue and do not completely eradicate bacterial biofilm from the root canal. The insufficient disinfection capability of root canal chemomechanical debridement advocated the development of more advanced root canal disinfection methods (Casal et al., 2009; Kishen, 2010; Siqueira et al., 2010).

Recently, nanoparticles, which are particles sized less than 100 nm, have demonstrated broad spectrum antimicrobial activity (Samuel and Guggenbichler, 2004). Biocompatibility was defined by Williams (2008) as the ability of nanoparticles to exert their biological activity without causing adverse reactions neither local nor systemic in the patients receiving the therapy. On the reverse, toxicity of NPs means their ability to disturb the normal physiology as well as damaging tissues and organs. Some studies showed that NPs could weaken the alveolar macrophage activity. For instance, silver NPs showed lung toxicity in the form of alveolar inflammation and granulomatous changes leading to a reduced pulmonary function (Sung et al., 2008). On the contrary, Chitosan is a non-toxic natural biopolymer of de-acetylated chitin that is of interest in the dental profession for its antimicrobial activity. Chitosan may also contribute to dentin remineralization by bonding to dentin calcium at its functional phosphate group. It is also a biodegradable, biocompatible broad-spectrum antimicrobial agent. Chitosan’s antimicrobial activity is affected by its solvent activity and molecular weight, and this activity increases at lower pH values (No et al., 2002; Xu et al., 2011; Li et al., 2012).

Over the last decade, ozone has been used in the medical field for its antiviral, antifungal and bactericidal activity as well as its high biocompatibility (Valacchi et al., 2005). Additionally, ozonated oils have been used to topically treat chronic ulcers and mucosal infections (Martinez-Sanchez et al., 2005). Ozonated olive oil exhibits high germicidal and oxygenating power against many microorganisms, favoring tissue healing and regeneration, and thus has been used on post-extraction alveolitis (Agapov et al., 2002).

Little is known about the efficacy of ozonated olive oil, silver nanoparticles or chitosan nanoparticles against microbial species isolated from persistent endodontic infections. The current study was conducted to evaluate the antimicrobial effect of these preparations against *E. faecalis, S. mutans*, and *C. albicans*, the predominant microbes in failed endodontic cases.

## Materials and Methods

### Test Materials

This study evaluated three test substances: an ozonated extra virgin olive oil (Novox^®^, MOSS S.r.l., Lesa – Novara, Italy) containing active oxygen in the form of peroxide ranged between 560–590 mmol-equiv/kg, prepared by introducing olive oil through a device generating ozone (Díaz et al., 2006); chitosan NPs (Ch-NPs) prepared by an ionotropic gelation technique (Sailaja et al., 2011) (NanoTech Egypt company for Photo-Electronics); and silver nanoparticles prepared by a chemical reduction method (Ratyakshi and Chauhan, 2009) (NanoTech Egypt company for Photo-Electronics).

Stock solutions (100 mg/ml) of Ag-NPs in sterile distilled water, Ch-NPs in 5% glacial acetic acid (HAC) and O_3_-oil in dimethylsulfoxide (DMSO) were prepared.

#### Test Microorganisms

Freeze-dried reference microorganisms, *S. mutans* ATCC 2419, *E. faecalis* OG1RF, and *C. albicans* MTCC 227, were used, which were obtained from the Department of Pharmaceutical Microbiology, Faculty of Pharmacy, Tanta University, Egypt.

#### Cell Line

Human Gingival Fibroblast cells (ATCC^®^ PCS-201-012^TM^) were obtained from the laboratory of the Tissue Culture Department of the Holding Company for Biological Products and Vaccines (VACSERA), Cairo, Egypt.

### Susceptibility of the Test Strains to Different Root Canal Medicaments

The antimicrobial activity of the tested root canal disinfectants was examined by determining the minimum inhibitory concentrations (MICs), minimum bactericidal concentrations (MBCs) and minimum fungicidal concentrations (MFCs) against endodontic pathogens using broth microdilution assay. The turbidity of each microbial suspension was adjusted to that of a 0.5 MacFarland standard then diluted in RPMI 1640 to give a final inoculum of 10^6^ CFU/ml. This inoculum was added to the wells of a 96-well microtitration plate containing twofold serial dilutions of the test medicament ranged from 0.039–40 mg/ml. Medium without inoculum was considered as a blank control. Inoculated medium without treatment was used as positive control. The wells were incubated for 24 h at 37°C, and then the OD_530_ was recorded using TECAN Sunrise^TM^ microtitre plate reader (Austria). MIC was defined as the lowest concentration of each medicament that inhibited the microbial growth compared to the untreated control culture. MBC was determined by performing viable count (CFU/mL) following 24 h of incubation at 37°C and was defined as the lowest medicament concentration that reduced the viable cells by more than 3 log10 steps (>99.9%) compared to the untreated control cultures. Independent assays were performed in triplicates (Gahlaut and Chhillar, 2013; Clinical and Laboratory Standards Institute [CLSI], 2016; Kaur et al., 2017). The antimicrobial synergy effect of different medicaments combinations was also evaluated *in vitro* using Checkerboard method as previously described by Tin et al. (2009). Briefly, for testing the combined effect of O_3_-oil and Ch-NPs, 96-well microtitre plate was used where the rows contained the same concentrations of O_3_-oil which was diluted twofold (from 20 to 0.3125 mg/ml) along the columns. The same concentrations of Ch-NPs are present in the columns which was diluted twofold (from 2.5 to 0.0391 mg/ml) along the rows. The wells were inoculated by the test pathogen at 10^6^ CFU/ml and the plates were incubated at 37°C for 24 h. For detecting the combined synergy effect, fractional inhibitory concentration (FIC) index was calculated as follows:

$$\begin{array}{c} {\mathrm{FIC}}_{\mathrm{index}}\;=\;\mathrm{FIC}\;A\;+\;\mathrm{FIC}\;B \end{array}$$

Where FIC A = MIC of drug A in the combination/MIC of drug A alone.

FIC B = MIC of drug B in combination/MIC of drug B alone.

The results were interpreted as synergism if the FIC index ≤0.5; additive effect if the FIC index of >0.5 and ≤4; and antagonism if the FIC index >4. The procedure was replicated three times. Similarly, the antimicrobial synergy effect of Ch-NPs/Ag-NPs and O_3_-oil/Ag-NPs was assessed by the same procedure.

### Cytotoxicity Analysis of the Test Medicaments on Human Fibroblast

The safety of the test compounds was evaluated on normal human fibroblasts. Briefly, approximately 100 μl of each of serially diluted compound was incubated with pre-cultured (6 × 10^4^ cell/ml) on 96-well plates. After 48 h, the cellular cytotoxic effects were quantified using the neutral red assay protocol (Borenfreund and Puerner, 1985). Half maximal inhibitory concentration (IC_50_) was calculated for each test medicament.

### Biofilm Assay Using Crystal Violet Quantitative Assay

The effect of the test medicaments on the biofilm biomass was evaluated by crystal violet binding assay (O’Toole, 2011). Overnight culture of each test strain was standardized to contain 10^6^ CFU/ml in trypticase soy broth. For generating dual or triple species biofilm, equal proportions of the standardized microbial suspensions were mixed together. About 100 μl of either of single, double or triple microbial suspension was dispensed to the corresponding well in a polystyrene 96-well plate containing the IC_50_ of the test compounds. Blank controls (medium without inoculum) were considered and inoculated medium without treatment was used as a positive control. After incubation at 37°C for 24 h, the planktonic cells were removed by aspiration of the spent media. All wells were washed twice using phosphate buffered saline solution (PBS) (pH 7.4) to remove loosely bound microbial cells and media components. Next, 125 μl of 0.1% crystal violet solution was added to each well to stain biofilm forming cells, incubated for 10 min at room temperature then rinsed three times with distilled water. For the quantitative assay, crystal violet was solubilized by adding 125 μl of 30% acetic acid to each well and incubated for 10 min at room temperature. The absorbance was measured for both the control and test groups by a plate reader at 550 nm with a reference filter of 570 nm.

### Effect of the Test Medicaments on Microbial Adhesion as a Virulence Factor Using Human Fibroblasts

Fibroblasts were cultured in Dulbecco’s Modified Eagle’s Medium (DMEM) (Lonza, United States) supplemented with 10% fetal bovine serum (FBS) (Lonza, United States) and 1% streptomycin/penicillin in 6-well plates (Greiner Bio-one, Germany) at approximately 1 × 10^5^ cells/ml then incubated at 37°C in a 5% CO_2_ atmosphere until they formed semi-confluent monolayers. Twenty-four hours post-incubation, the exhausted media were replaced with fresh DMEM. Overnight cultures of the test microbes were standardized to a final inoculum of 10^7^ CFU/ml using 0.5 MacFarland standard and confirmed by viable count. Approximately 50 μl of each standardized microbial cell cultures, pretreated with non-toxic doses of the medicaments, were incubated at 37°C for 3 h. After incubation, the medium was discarded and the wells were washed three times with PBS to remove the unbound microbial cells. One milliliter of methanol was added to each well for 1 min, then replaced with 4 ml of freshly diluted Giemsa stain (1:10) and incubated for 30 min at room temperature. The stain was discarded, and the fibroblast monolayers were washed sequentially with tap water, acidified water (1 ml concentrated H_2_SO_4_ in 1 L of tap water) and tap water again. The wells were examined at 20× magnification under a light microscope (Olympus, Carlsbad, CA, United States). The microbial strains were considered adherent if they formed characteristic microcolonies on >40% of the fibroblast cell surface. The data represented at least three trials (Cravioto et al., 1979; Presterl et al., 2003).

### Antibiofilm Challenge of the Test Medicaments Against Endodonotic Pathogens Using an *ex Vivo* Premolar Teeth Model

Among the tested medicaments, Ch-NPs and O_3_-oil were selected due to antibiofilm activity at their IC_50_. The effect of the later medicaments, either separate or combined together, was evaluated on the mature multi-species biofilm *ex vivo* using extracted premolar teeth. The effect of DMSO or 5% HAC, as vehicles, on the microbial biofilm was also assessed.

### Preparation of the Extracted Premolar Teeth

A total of 168 intact single rooted human premolars with single straight root canals extracted for periodontal or orthodontic reasons were collected after obtaining the patients’ written consent. This was based on the protocol approved by The Research Ethics Committee at the Faculty of Dentistry, Tanta University, Egypt. The teeth were collected in 2.5% sodium hypochlorite solution then mechanically cleaned and decoronated under water coolant using a diamond disk (Edenta AG, AU/SG, and Switzerland). The step-back technique was used to chemomechanically debride the canals using stainless steel k-files (Dentsply/Maillefer, Ballaigues, Switzerland) and 2.5% sodium hypochlorite as an irrigant with a master apical file size 40.

The apical 3 mm as well as crown (2–3 mm) of each root specimen was cut using the diamond disk to obtain 12 mm-long standardized root specimens. The canal lumen for each specimen was prepared to a standardized size by passing rotating peeso drills, sizes 1 and 2, from the coronal end until just visible at the apical end. A 2 mm-deep cavity was prepared on the apical ends of each canal using #4 slow speed round bur (Dentsply/Maillefer) and sealed with acrylic resin to prevent micro leakage. The prepared root canals were conditioned with 10 mL of 17% EDTA (Canal +, Septodont, France) for 60 s to remove the smear layer, washed with 10 mL of 5.25% NaOCl, flushed with 10 mL of saline solution and dried with paper points (Dentsply-Maillefer, Ballaigues, Switzerland). Teeth were subjected to sterilization by steam autoclave for 15 m at 121°C (Samuel and Guggenbichler, 2004; Gomes-Filho et al., 2010).

### Generation of Mature Biofilm in Premolars

A single colony from the overnight cultures of each microbial strain was suspended in sterile brain heart infusion broth to prepare 10^6^ CFU/ml using the 0.5 McFarland standard. Equal portions of each microbial suspension were mixed together in a Falcon tube to be used for generating multiple species biofilm. Premolar teeth were aseptically transferred to the tube containing the mixed microbial suspension, then incubated under anaerobic conditions at 37°C for 1 week to obtain a mature multi-species biofilm. The culture broth was refreshed every 2nd day to ensure bacterial viability and remove dead cells. At the end of the incubation period, specimens were aseptically removed from the tubes and washed with sterile PBS to remove unbound microbes and culture media. Another Falcon tube contained teeth in a medium without inoculation was used as negative control (Flemming and Wingender, 2010).

### Application of Different Medicaments Into Biofilm Containing Premolars

Fifty six teeth were divided into eight groups (*n* = 7) to test the three different intracanal medicaments including; O_3_-oil, Ch-NPs and a combination of both. The control group (G.4, *n* = 7) was inoculated and kept untreated in the incubator for the remainder of the test. The negative control group (G.5, *n* = 7) in which the medium was free from inoculum and treatment to exclude contamination throughout the experiment. Vehicles (DMSO or 5% HAC) were also tested to exclude their effect on mature biofilm (G.6 and G.7, respectively). The positive control group (G. 8) which test calcium hydroxide suspension. Test samples were irrigated with sterile saline for 2 m and dried with paper points. The test medicaments were injected into the corresponding root using syringe and canal orifices, then aseptically sealed with modeling wax (Cavex, Holland). The specimens were transferred into sterile petri dishes and covered with humid sterile gauze. The plates were incubated at 37°C for 1 week and evaluated daily for viability of microbial cells (Flemming and Wingender, 2010). This experiment was repeated three times.

### Quantitative Determination of Viable Microbes in the Biofilm

Viable microbes were quantified in the biofilm post-treatment. The wax seal was removed from the canal orifice of one root from each group daily. Each canal was flushed with sterile saline solution along with light circumferential filing using the master apical file to remove intracanal medicament. Root canals were dried with sterile paper points.

A 400 μm-deep sample from the dentin lining of each canal was collected by passing a rotating size 4 peeso drill from the coronal to the apical ends of each sample. Dentin debris obtained from the canal walls were immediately collected onto separate Eppendorf tubes to a weight of 10 mg and serially diluted. The harvested dentin debris was added to 1 ml of sterile saline, then vortexed for 30 s to collect the microbes on the dentin debris. Each sample was serially diluted twofold for five dilutions, then 10 μL of each specimen were plated out on brain heart infusion agar plates and incubated at 37°C for 24 h. Viable microbes were counted and represented as colony-forming units (CFU/ml). This procedure was repeated three times. The CFU values were converted to log_10_ and the mean log values with standard deviations (SD) were calculated (Flemming and Wingender, 2010).

### Statistical Analysis

The data were analyzed using IBM SPSS statistics for Windows, version 21.0 (IBM Corp., Armonk, NY, United States). One-way ANOVA, and Tukey’s test at α = 0.05 were used to determine significant differences between the groups. Graph Pad Prism was used to calculate the IC_50_ from the plotted curves of the cytotoxicity assay.

## Results

Broth microdilution assay revealed that all of the test medicaments had high MIC and MBC values as recorded in **Table 1**. Ch-NPs exerted 4- and 8-fold increases in the microbicidal activity compared to O_3_-oil against *C. albicans*, and both of *E. faecalis* and *S. mutans*, respectively. Synergism (FIC index ≤ 0.5) was recorded for O_3_-oil/Ch-NPs combination when it was assessed for the antimicrobial synergy *in vitro* against the test endodontic pathogens. Moreover, an additive effect (FIC index of >0.5 and ≤4) was noticed when O_3_-oil/Ag-NPs and Ch-NPs/Ag-NPs combinations were tested as recorded in **Table 2**.

**Table 1:** Determination of the MIC, MBC and MFC of the three medicaments against test strains.

Medicaments test strains	MIC, MBC, and MFC of the test medicaments in (mg/ml)					
	Chitosan		Silver nitrate		Ozonated olive oil	
	MIC	MBC or MFC	MIC	MBC or MFC	MIC	MBC or MFC
*E. faecalis*	0.625	1.25	5	5	10	10
*S. mutans*	0.625	1.25	5	5	10	10
*C. albicans*	0.625	1.25	5	5	5	5

**Table 2:** FIC indices for different combinations of the test root canal medicaments against persistent endodontic pathogens.

Medicament combination	FIC indices of the combinations against test pathogens		
	*E. faecalis*	*S. mutans*	*C. albicans*
O3-oil/Ag-NPs	0.509	0.564	0.565
O3-oil/Ch-NPs	0.115	0.194	0.206
Ch-NPs/Ag-NPs	0.732	0.548	0.791

**FIGURE 1:**
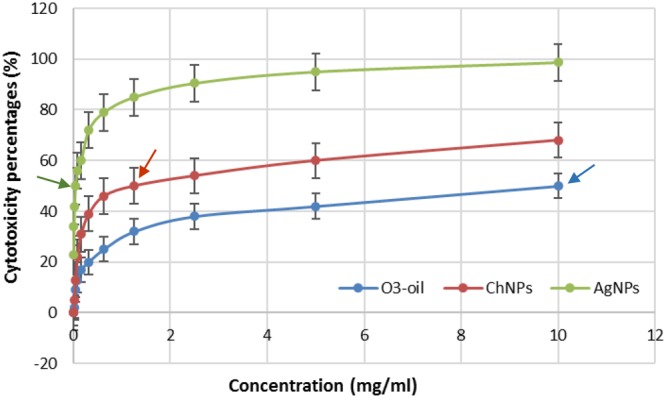
Cytotoxicity test of Ch-NPs, O_3_-oil, and Ag-NPs on human fibroblast. Arrows point to the IC_50_ of each medicament.

The neutral red assay results for testing cytotoxicity on human fibroblasts are shown in (**Figure 1**). The IC_50_ for the three test disinfectants was calculated from the plotted curves and ranged from 0.039–10 mg/ml. One-way ANOVA revealed a significant difference (*p* < 0.001) between the test groups and the untreated control group. The IC_50_ of O_3_-oil was the safest, followed by the Ch-NPs (1.25 mg/ml).

**FIGURE 2:**
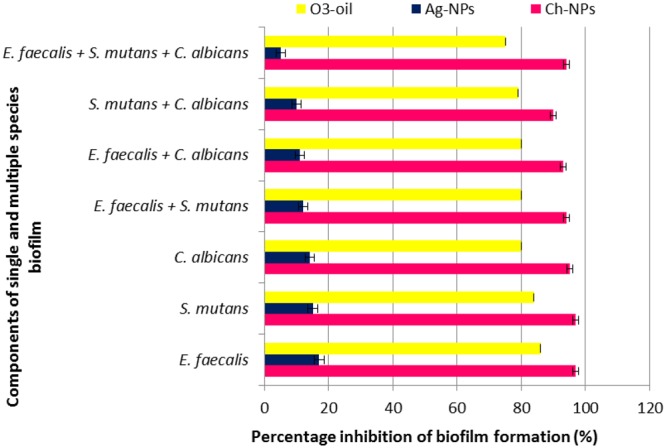
Effect of the three tested medicaments on the single and mixed species biofilm.

Effect of the test medicaments on the formation of single or mixed microbial species biofilms was evaluated by a crystal violet assay that measures only biomass by binding of positively charged CV to all negatively charged residues in the biofilm. The Ch-NP solutions had the lowest mean value of absorbance, followed by O_3_-oil then the Ag-NP solutions, while the control group had greatest mean value (**Figure 2**). Ch-NPs reduced single and mixed species biofilm by 97 and 94%, respectively. Furthermore, O_3_-oil exerted 86 and 79% reduction in biofilm, respectively. However, Ag-NPs showed non-significant reduction. Notably, the control group was significantly different from the other groups.

**FIGURE 3:**
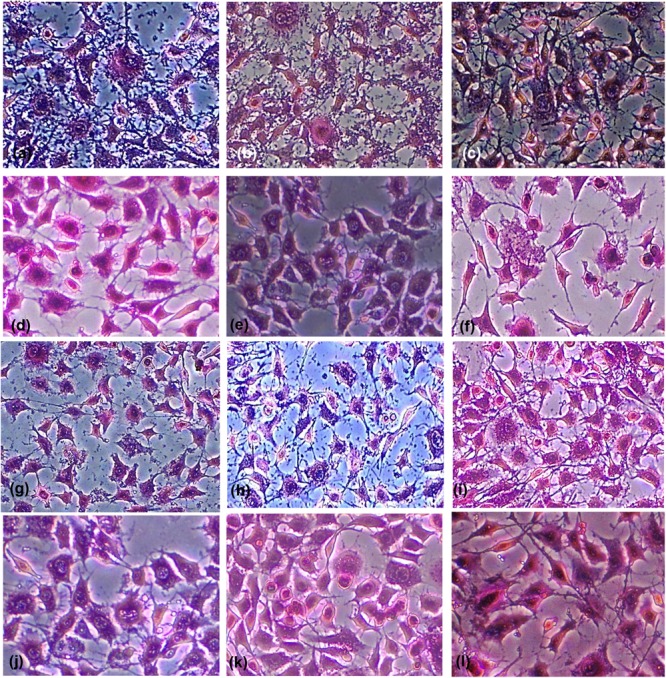
Light micrographs of Giemsa-stained untreated fibroblast cells showing adherence of **(a)** *Enterococcus faecalis*, **(b)** *Streptococcus mutans*, and **(c)** *Candida albicans*, to the cells a swell as the polystyrene of the microtitration plate. The light micrographs **(d–f)** show cells treated with Ch-NPs, **(g–i)** treated with Ag-NPs, **(j–l)** treated with O_3_-oil. Magnification 20×.

**FIGURE 4:**
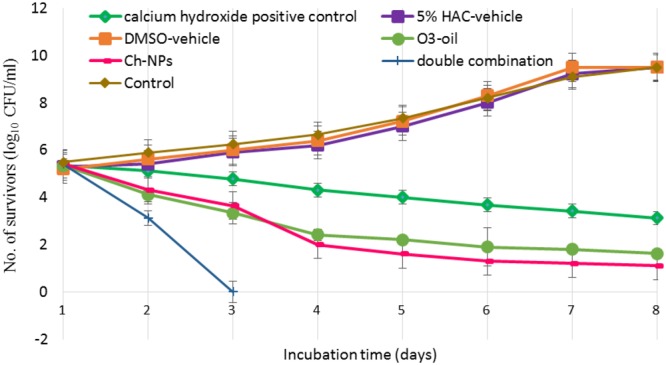
Time-kill curves showing the number of microbial survivors recorded day to day in the debris of the dentinal tubules at 400 microns to assess the effect of O_3_-oil, Ch-NPs, alone or in combination on the mature biofilm developed for 1 week. Data represent mean ± standard deviation.

Antiadherent test was assessed using Giemsa staining and microscopy (**Figure 3**). It revealed that the Ch-NPs and O_3_-oil were the most effective at reducing the fibroblast-microbial cell adhesion by reducing the number of fibroblast-associated microbes to almost zero (*p* < 0.05) compared to the untreated microbial cells (control), which showed high adhesion indices. Non-significant reduction in fibroblast-bacterial cell adhesion was also noted with Ag-NPs. Moreover, it had less effect on *C. albicans*-fibroblast adhesion (**Table 3**). The time-kill kinetics for the different test treatments showed that killing was time-dependent throughout the 7-day incubation with the mature biofilm. ANOVA revealed a significant difference between groups (*p* < 0.05). The fastest kill rate was recorded for the double combination (O_3_-oil/Ch-NPs), as it significantly reduced (6-log reduction) the number of survivors by the 2nd day of treatment. The separate medicaments had slower kill rates than the combined one (**Figure 4**). There were no significant effect for DMSO or 5% HAC on the microbial biofilm as compared to the untreated control group (**Figure 4**).

**Table 3:** The mean values of the associating microbial cells to the fibroblast or the polystyrene surface as well as the number of fibroblast associated with the microbial cells, in the presence or absence of the test medicaments.

Groups		Control			Ch-NPs treated cells			Ag-NPs treated cells			O_3_-oil treated cells		
Strain code		*E. faecalis*	*S. mutans*	*C. albicans*	*E. faecalis*	*S. mutans*	*C. albicans*	*E. faecalis*	*S. mutans*	*C. albicans*	*E. faecalis*	*S. mutans*	*C. albicans*
Associated fibroblast		100 ± 0.33^∗^	98 ± 0.27^∗^	100 ± 1.5^∗^	0^∗^	0^∗^	0^∗^	82 ± 0.34	90 ± 0.23	95 ± 0.11	0^∗^	0^∗^	7 ± 1.8^∗^
Microbial cell/polystyrene surface association	Mean ± Standard deviation	150 ± 0.73^∗^	200 ± 0.22^∗^	110 ± 0.14^∗^	0^∗^	0^∗^	0^∗^	122 ± 0.11	185 ± 0.08	100 ± 0.36	0^∗^	0^∗^	0.45^∗^ ± 0.06
Associating microbes		400 ± 0.25^∗^	389 ± 0.13^∗^	420 ± 0.11^∗^	0^∗^	0^∗^	0^∗^	380 ± 0.15	380 ± 0.07	411 ± 0.05	0^∗^	0^∗^	10 ± 0.24^∗^

*^∗^P****<****0.05, student’s test.*

## Discussion

Microbial biofilm is a survival mechanism in which microbes form a strong shield against chemotherapeutic attack (Flemming and Wingender, 2010). Pulpo-periapical diseases are biofilm-mediated infections that are difficult to treat with conventional antimicrobials (Römling and Balsalobre, 2012). The physicochemical environment of the biofilm is markedly modified by *C. albicans*, which produces β-1,3 glucan, providing stability to the biofilm’s 3D matrix. In addition, extracellular polymeric substance (EPS) secretion helps other pathogens, such as *S. mutans*, adhere and form microcolonies that enable growth in the presence of *C. albicans*. The acidity of the biofilm increases as it matures in the presence of acidogenic pathogens such as *S. mutans*. The coexistence of *E. faecalis* with these pathogens stimulates production of superoxides that trigger hyphae formation in *C. albicans*. This results in a more complex biofilm due to the complexity of the bacterial-fungal association (Xiao et al., 2012; Falsetta et al., 2014). Accordingly, innovative approaches are necessary to prevent and eradicate biofilm formation. This study was conducted to assess the antimicrobial, antibiofilm and antivirulent efficacy of Ch-NPs, Ag-NPs, and O_3_-oil against the *S. mutans, E. faecalis*, and *C. albicans* bacterial-fungal biofilm that is encountered in many resistant endodontic infections. The three test disinfectants, along with a novel mixture, were assessed in an *ex vivo* model of infected root canal premolar teeth specimens.

The antimicrobial effect of Ch-NPs, Ag-NPs, and O_3_-oil on *S. mutans, E. faecalis*, and *C. albicans* was evaluated by routine microbiological techniques such as MIC, MBC, and MFC tests. Aliasghari et al. (2016) reported that 5 mg/ml of Ch-NPs inhibited the growth of *S. mutans*, showing an inhibition zone diameter of 15 mm. Berenji et al. (2014) stated that O_3_-oil had powerful bactericidal and fungicidal activity. They reported that the MIC of ozonated olive oil was 233.33 mg/ml against *C. albicans*. This indicates that our preparation was 47 times more effective. Additionally, Krishnan et al. (2015) stated that the optimum MIC of Ag-NPs against *E. faecalis* was 5 mg/ml, similar to that of the current study. Conversely, Lotfi et al. (2011) reported that the MIC was 50 μg/ml, and this variation may be due to differences in the Ag-NPs preparation methods as well as particle size. A synergistic effect was recorded for O_3_-oil/ChNPs combinations against endodontic pathogens with FIC index ≤ 0.5 giving a strong evidence on the better combination of both candidates against the test microbes. A similar result was reported by Tin et al. (2009).

Nanotechnology has gained a great interest due to several health benefits and industrial applications such as drug delivery, cancer treatment, gene therapy and medical imaging. Although nanoparticles has many advantages, nanotoxicity to human and environment gained the attention of the researchers (Ray et al., 2009). These smaller materials might be biodegraded within the cell resulting in their accumulation leading to intracellular changes such as alterations of genes. Argyria is a condition in which the skin is blue–gray colored due to the exposure to high levels of silver NPs, however, the low levels of this NPs might result in breathing problems and allergic reactions (Ray et al., 2009). The study conducted by Burd et al. (2007) tested the cytotoxicity effect of commercially available dressings containing Ag-NPs in a cell culture monolayer. Their study showed that the cytotoxicity was concentration dependent. However, Hernández-Sierra et al. (2011) and Soleimani et al. (2015) reported that Ag-NPs (80–100 nm) showed no apparent cytotoxicity on fibroblasts due to the low concentration. In contrast, the cytotoxicity results in our investigation showed that the most cytotoxic effect was exerted by the Ag-NPs, while the safest effect was reported for O_3_-oil. This high cytotoxicity of the Ag-NPs was confirmed by previous studies that related this to the toxicity from cytochrome C release, together with the induction of reactive oxygen species, which produce oxidative stress leading to a series of cellular events, including inflammation, membrane lipid peroxidation, apoptosis and DNA damage (Gomes-Filho et al., 2010; Kishen, 2010; Guzman et al., 2012). On the reverse, chitosan was previously approved by FDA as safe polymer (Dash et al., 2011). Additionally, several non-specific enzymes could degrade it. Furthermore, it is able to stabilize the membranes of RBCs and hence it is hemostatic (Aranaz et al., 2010). Similarly, FDA allowed the use of ozonated olive oil on food to fight bacteria. It has also a positive impact on tissue repair and wound healing due to its potent anti-inflammatory effect on the NF-kappa B system and the ability to induce fibroblast proliferation confirming its safety when reach the systemic circulation (Ramzy et al., 2005). Therefore, we used the IC_50_, safe on the fibroblast, for all medicaments to be tested as anti-biofilm and anti-adherent.

One of the most important problems in treating endodontic disease is the infectious process of the biofilm formation (Jhajharia et al., 2015). In the current study, CV assay was used to measures the biomass by binding of positively charged CV to all negatively charged residues in the biofilm. Both of Ch-NPs and O_3_-oil significantly (*P* < 0.05) reduced the total biofilm mass compared to the control groups, specifically the Ch-NPs, which showed 97 and 94% reductions for single and mixed species biofilms, respectively. This agrees with the results of Costa et al. (2014), who reported that Ch-NPs significantly reduced biofilm formation in *C. albicans* and *S. mutans* up to 92.5 and 93.4%, respectively. This supports the finding that our medications acted against the biofilm. The slightly lesser effect of the treatments on the mixed species biofilm in this study can be explained by the findings of Bachtiar et al. (2016), who stated that *C. albicans* was resistant in the presence of the encapsulated *E. faecalis* ATCC 29212 biofilm at the intermediate stage (24-h). *E. faecalis* produces peptidoglycan that induces hyphal growth in *C. albicans*. Consequently, this stimulatory effect at the intermediate stage of biofilm development indicates the ability of *E. faecalis* and *C. albicans* to cohabitate. Additionally, Huycke et al. (1998) stated that *E. faecalis* produces extracellular superoxides that can trigger reactive oxidative stress, inducing filamentation in *C. albicans*. However, this relationship can become antagonistic with unencapsulated *E. faecalis* isolates in mature biofilm. Similarly, *S. mutans* can promote *C. albicans* biofilm due to their mutualistic relationship (Falsetta et al., 2014). However, the interaction between *E. faecalis* and *S. mutans* is variable and strain-dependent (Li et al., 2014).

The first step in biofilm formation depends on the microbe’s ability to adhere to solid surfaces (Shen et al., 2011). One of the mechanisms of action of our treatments was as an antibiofilm. In this work, 1.25 mg/ml of Ch-NPs completely inhibited all test microbes from adhering to normal human fibroblasts as well as to the polystyrene surface of microtitration plates. Aliasghari et al. (2016) reported that 5 mg/ml of Ch-NPs reduced the adhesiveness of cariogenic *S. mutans* by 93.4%, indicating the effectiveness of our medicine. Additionally, Di Giulio et al. (2013) reported that the chitosan-silver mixture reduced the adherence of streptococci to polystyrene surfaces.

In our study, mixed species mature biofilm was developed in premolars to simulate clinical cases. The effect of the medicaments on mature biofilm in premolars was evaluated using viable counts of CFU/ml to test their viability in biofilm within the dentinal tubules. The O_3_-oil/Ch-NPs combination worked the best, exerting a significant killing effect in the form of a 6-log reduction in viable cells (i.e., killing 99.9999% of the viable microbes in 2 days). It worked faster than the other treatments and the traditional medications that need at least 1 week to provide acceptable results. Calcium hydroxide was used as a positive control in our study. It was less effective than O_3_-oil, Ch-NPs, and their combination. This result was supported by the findings of Upadya et al. (2011) who reported that calcium hydroxide was ineffective for treatment of root canal infections forming either single of multispecies biofilms. Del Carpio-Perochena et al. (2017) mentioned that a combination of calcium hydroxide and Ch-NPs could potentiate the killing of bacteria forming the biofilm within 7–14 days. Moreover, Devaraj et al. (2016) stated that the triple antibiotic paste was more effective than calcium hydroxide against *E. faecalis* biofilm. Valacchi et al. (2005), who reported that ozonated olive oil had the strongest effect on mature biofilm. The oil’s potent antimicrobial activity is attributed to the stabilization of ozone as ozonide in addition to other products including peroxide, hydroperoxide, diperoxide, polyperoxide and aldehydes, which are generated by the interaction between ozone and olive oil (Pryor and Uppu, 1993). Di Giulio et al. (2013) reported that chitosan nanocomposite system exerted similar result on biofilm. Chitosan antibiofilm activity can be explained by several mechanisms, among which is its ability to penetrate and damage the biofilm due to its cationic properties (Carlson et al., 2008). A recent study reported that using light-activated curcumin as an antibiofilm killed the most *E. faecalis* cells at 200 and 400 micron depths compared to the control; however, this did not differ significantly from the triple antibiotic paste (Devaraj et al., 2016).

In view of the current *in vitro* study results, further *in vivo* investigations are recommended to determine the best concentration, and application time for each medicament, as well as the optimum ratio of the double combination, to obtain the best antimicrobial effect with the least adverse reactions.

Based on the current study, ozonated olive oil had the least cytotoxic effects, while chitosan nanoparticles showed better antimicrobial activity against the tested endodontic pathogens when used at its MIC and MBC/MFC compared to both silver nanoparticles and ozonated olive oil. Our tested double combination has the potential to prevent microbial biofilm formation and eradicate mature mixed-species biofilms. Incorporating the tested medicaments, either separately or in the novel double combination, into intracanal treatments, root canal sealers and irrigating solutions can prevent biofilm formation and eradicate resistant endodontic pathogens from the root canal, thereby increasing the success rate of endodontic treatment. These medicines might also be valuable if added to toothpastes and/or mouth rinses to combat harmful oral microbial biofilms.

## Author Contributions

LA-M and NE-D conceived the experiments. LA-M, NE-D, and WG conducted the experiments. LA-M and NE-D analyzed the results. All authors wrote and reviewed the manuscript.

## Conflict of Interest Statement

The authors declare that the research was conducted in the absence of any commercial or financial relationships that could be construed as a potential conflict of interest.
